# Drug Resistance in Glioblastoma: The Two Faces of Oxidative Stress

**DOI:** 10.3389/fmolb.2020.620677

**Published:** 2021-01-27

**Authors:** Christophe Olivier, Lisa Oliver, Lisenn Lalier, François M. Vallette

**Affiliations:** ^1^Faculté des Sciences Pharmaceutiques et Biologiques, Nantes, France; ^2^Université de Nantes, INSERM, CRCINA, Nantes, France; ^3^CHU de Nantes, Nantes, France; ^4^LaBCT, ICO, Saint Herblain, France

**Keywords:** glioblastoma, oxidative stress, drug resistance, tumor microenvironment, nutrition

## Abstract

Glioblastomas (GBM) are the most common primary brain tumor with a median survival of 15 months. A population of cells with stem cell properties (glioblastoma stem cells, GSCs) drives the initiation and progression of GBM and is localized in specialized microenvironments which support their behavior. GBM are characterized as extremely resistant to therapy, resulting in tumor recurrence. Reactive oxygen species (ROS) control the cellular stability by influencing different signaling pathways. Normally, redox systems prevent cell oxidative damage; however, in gliomagenesis, the cellular redox mechanisms are highly impaired. Herein we review the dual nature of the redox status in drug resistance. ROS generation in tumor cells affects the cell cycle and is involved in tumor progression and drug resistance in GBM. However, excess ROS production has been found to induce cell death programs such as apoptosis and autophagy. Since GBM cells have a high metabolic rate and produce high levels of ROS, metabolic adaptation in these cells plays an essential role in resistance to oxidative stress-induced cell death. Finally, the microenvironment with the stromal components participates in the enhancement of the oxidative stress to promote tumor progression and drug resistance.

## Introduction

Glioblastoma (GBM, WHO classification IV grade) covers about 54% of all gliomas and 16% of total brain tumors (Louis et al., 2016). It is the most common primary brain tumor with an incidence of approximately three cases per 100,000 inhabitants in France. Its prognosis is bleak: the average survival is 15 months once the diagnosis is established (Ostrom et al., 2015), with <10% of patients surviving 5 years after diagnosis. GBM is characterized by inter- and intratumoral heterogeneity, which has been suggested to contribute to resistance to treatment. As such, relapse may occur, in part due to a therapy-resistant subpopulation of GBM stem cells (GSCs) present or by the induction of dedifferentiation in the non-GSC subpopulation due to alterations in the REDOX state due to therapy (Bao et al., 2006; Diehn et al., 2009). The standard therapy follows the Stupp protocol (Stupp et al., 2005), which consists of tumor surgical resection followed by post-operative ionizing radiation (IR), comprising 60 Gy/30 fractions and concomitant plus adjuvant temozolomide (TMZ) chemotherapy. However, due to the infiltrating nature of GBM, complete removal of the tumor is not always possible. Chemotherapy and IR share common pathways to cell death, inducing DNA damage either directly or indirectly by generating reactive oxygen species (ROS). It is accepted that while low levels of ROS enhance cell growth and differentiation, higher levels induce cell death. Thus, in many cancers, the level of ROS is an important marker of the state of the tumor.

Numerous studies have demonstrated the presence in GBM of a subpopulation of self-renewing and pluripotent GBM stem-like cells (GSCs) responsible for GBM formation, maintenance, invasiveness, and recurrence (Bao et al., 2006). Tomasetti et al. (2017) have shown that neural stem cells (NSCs) in the subventricular zone of the human brain might contribute to GBM formation and development, and Lee et al. (2018) have demonstrated that the somatic driver mutations in these NSCs have the ability to stimulate the development of the tumor. In addition, Chen et al. (2012) have identified a relatively quiet subpopulation of GBM cells, with properties similar to cancer stem cells that are the source of new tumor cells post-chemotherapy treatment and could be responsible for sustaining long-term tumor growth. A hierarchy in the stem cell population was likewise shown, and chemotherapy could assist in the expansion of pre-existing drug-resistant GSCs (Lan et al., 2017). Moreover, glial cells reside in a specific tumor microenvironment (TME), which supports tumor growth via direct contact and secretion. The TME participates in tumorigenesis by generating a ROS niche through oxidative stress (OS) (Schiffer et al., 2018). OS enhances cancer cell invasiveness but also supports GSC maintenance (Janiszewska et al., 2012) and thus participates in the evasion/resistance to treatment and consequently recurrence of the tumor (Burdon et al., 1990; Costa et al., 2014). Furthermore, another role of ROS in TME would be to influence, quantitatively and qualitatively, the nature of the infiltrating non-cancer cells in the tumor (Weinberg et al., 2019).

**Figure 1:**
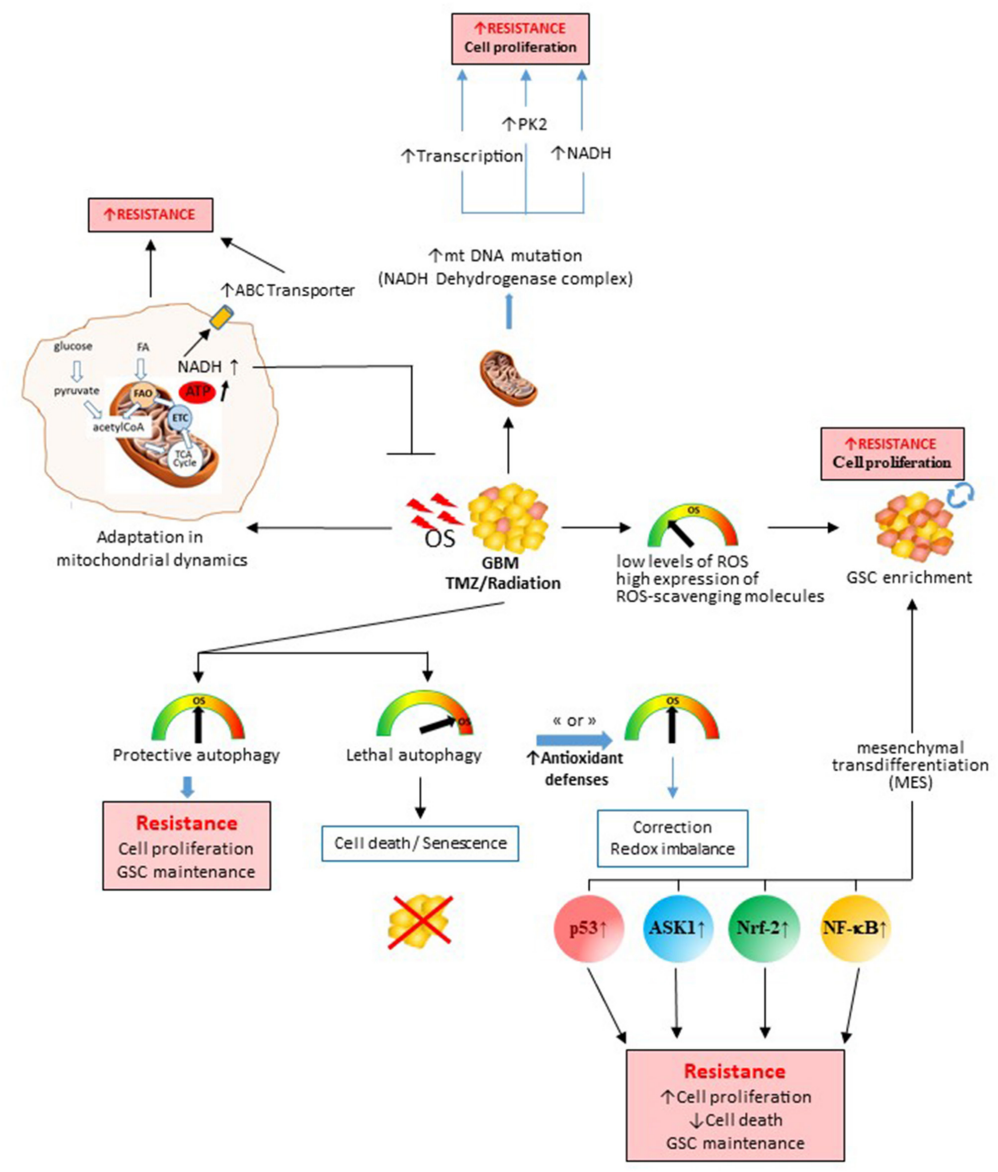
Role of therapy-induced oxidative stress (OS) in the genesis of therapeutic resistance processes and tumor escape. OS induced by chemo- or radio-therapy during the treatment in glioblastomas will impact the survival/death balance, cell metabolism, glioblastoma stem cell subpopulation, and mitochondrial DNA. All these induced mechanisms will promote therapy escape and tumor recovery. Depending on the degree of stress induced and the antioxidant defenses activated, the cancer cell will either die or survive by different processes. ASK1, apoptosis signal-regulating kinase 1; NADH, reduced form nicotinamide adenine dinucleotide; NF-κB, nuclear factor kappa-light-chain-enhancer of activated B cells; Nrf-2, nuclear factor erythroid-2-related factor 2; PK-2, pyruvate kinase.

In this review, we will discuss the role of OS in radio- and chemotherapy in GBM. A listing of the activation of common cellular stress pathways, in particular with the production of ROS and engendering of metabolic reprogramming, is summarized in Figure 1. We will also discuss how the stress induced by the therapy regimes participates in the selection of GSCs and/or enhances the dedifferentiation of non-GSCs to GSCs caused by redox modifications. In addition, we will describe how, during gliomagenesis as well as during the local response to therapy, the TME contributes to stress and helps cancer cells escape through OS.

## Oxidative Stress and Cancer

Two different hypotheses described the effects of ROS in tumor cells, the first being “the threshold concept for cancer therapy,” which states that as the amount of ROS in cancer cells increases, the ratio between ROS and the antioxidants is maintained in a well-controlled steady equilibrium, after which any further augmentation in ROS or reduction in antioxidants would result in cell death or increased sensitivity to therapy (Kong et al., 2000). The second hypothesis suggests that when both tumor and normal cells are subjected to comparable levels of exogenous ROS-producing agents, the intracellular ROS levels of tumor cells increase more readily than normal cells to attain a threshold and induce cell death (Wang and Yi, 2008). The changes induced during tumorigenesis are represented as changes in the redox status in tumor cells, generally activating the creation of ROS. These ROS molecules are typified as oxygen-carrying molecules that have reactive properties, consisting of radicals comprising ${\text{O}}_{2}^{-}$ (superoxide) and HO^•^ (hydroxyl) as well as non-radicals such as H_2_O_2_ (hydrogen peroxide). ROS originate from oxygen, which is used in numerous metabolic reactions in organelles such as the mitochondria, endoplasmic reticulum (ER), and peroxisomes. On a physiological level, the action of ROS is to regulate signal transduction pathways and moderate the activity of mitochondrial enzymes and transcription factors. In cancer, the general consensus is that an elevated production of ROS would engender tumorigenesis by impeding DNA repair mechanisms, resulting in an accumulation in DNA damage, including base modifications, inter- and intra-strand binding, and DNA–protein bonds, as well as an increase in cell proliferation due to the increase in H_2_O_2_ and ${\text{O}}_{2}^{-}$. Indeed OS can generate ROS-induced damage in proteins, lipids, and DNA, resulting in genomic instability. As such, cancer cells are constantly maintaining a balance between the response to OS and ROS production for their survival (Kong et al., 2000). The resilient cellular response to low oxygen concentrations under both physiological and pathological conditions involves varied pathways; the best known are the hypoxia-inducible factors (HIFs) and the ER stress responses. The HIF-related mechanisms respond to alterations in oxygen concentration that would influence the ability of GSCs to initiate tumors. Indeed GSCs respond to hypoxia, and an augmentation in the level of HIFs was detected in GSCs. Moreover, multiple HIF-regulated genes are selectively expressed in GSCs in comparison to other GBM cells (Li et al., 2009). Hypoxia has been shown to promote the self-renewal capacity of GSCs as well as the ability to promote a more stem-like phenotype in the non-stem cell population by the up-regulation of important stem cell factors including Oct4 or Nanog (Heddleston et al., 2009).

HIF-2α is likewise expressed in GSCs at oxygen levels comparable to normal *in vivo* levels (2–5%). Hence, in highly heterogeneous GBM tumors, different tumor cells survive in a wide spectrum of oxygen concentrations in their environment and, because of the levels of HIFs, generate a gain over normal cells by adapting to their surroundings. By consequence, the enrichment of ROS occurs, harming non-malignant cells and resulting in apoptosis, while tumor cells would survive and prosper in a hypoxic microenvironment.

## The Intracellular Sites of ROS Genesis

The main sources of ROS production in tumor cells include NADPH oxidases (NOXs) and the electron transport chain (ETC) in the mitochondria. Additionally, the ER also produces important amounts of ROS from oxidoreductases and NOXs. Both the mitochondrial ETC and NOXs decrease oxygen to the reactive superoxide anion (${\text{O}}_{2}^{-}$), which undergoes a complex sequence of conversion reactions that result in the formation of hydrogen peroxide (H_2_O_2_) as well as hydroxyl radical (^−^OH) or reactive nitrogen species (RNS), including nitric oxide (NO^−^). Besides the ETC, the mitochondria also contain enzymes that produce ROS. Some examples of mitochondrial enzymes that produce ROS are the inner mitochondrial membrane dihydroorotate dehydrogenase that is coupled to complex III of the respiratory chain and generates ${\text{O}}_{2}^{-}$ and H_2_O_2_, glycerol-3-phosphate dehydrogenase 2 (GPDH-2), which produces ROS via the reverse electron transport from flavin adenine dinucleotide (FAD) to the electron transfer chain, and the outer mitochondrial membrane monoamine oxidase (MAO) that releases ROS through the deamination of serotonin to catecholamine. About 2% oxygen is used by the mitochondria to generate ${\text{O}}_{2}^{-}$, and thus this organelle is considered as the main source of ROS and this through multiple pathways (Murphy, 2009). As stated previously, the ER is another organelle that participates in ROS production in the cell and encompasses two important sources of ROS: the first is NOX4, a member of the NADPH oxidase family, and the second is the Ero1α-PD-I protein-folding pathway. Thiol-disulfide exchange reactions between the catalytically active domains of PD-I and cysteines of nascent client proteins could result in rupture, creation, or isomerization of disulfide bonds. This oxidoreductase reaction necessitates the activity of the membrane-bound ER oxidoreductin 1α (Ero-1α), a FAD-dependent oxidoreductase. A derivative of this Ero-1α-PD-I oxidative protein folding pathway is H_2_O_2_, which would enhance the oxidative environment in the ER, which is characterized by a high glutathione disulfide-to-glutathione (GSH) ratio. The folding of oxidative proteins involves quiescin-sulfhydryl oxidase 1 (QSOX-1) that produces H_2_O_2_ and contributes in the creation of disulfide bonds in proteins.

## Bypassing Mechanisms of Chemo- and Radio-Resistance in Glioblastoma

Morphologically, GBM tumors contain chronic hypoxic regions (Rong et al., 2006; Matschke et al., 2016), with a naturally high resistance to treatment due to an augmentation in hypoxia-inducible factor-1 alpha (HIF-1α) (Hsieh et al., 2012). In these regions, the concentration of H^+^ increases with distance from the blood vessels due to the distance of diffusion and the increased production of lactate from anaerobic cells, creating a highly acidic region (Fang et al., 2008), which is also linked to radio-resistance (Raghunand and Gillies, 2000; Hirschhaeuser et al., 2011), while lactate would act as an antioxidant by scavenging ${\text{O}}_{2}^{\cdot -}$ and OH^·^, thereby inhibiting lipid peroxidation (Groussard et al., 2000). In these regions, dormant cells and hypoxic cells have gene profiles distinct from cells found in well-vascularized regions, which is associated with a lower drug sensitivity (Lu et al., 2010). A subpopulation of GSCs resides in these hypoxic niches far from blood vessels due to their ability to adapt to the low O_2_ microenvironment (Ito and Suda, 2014). Consequently, structural tumor hypoxia or a hypoxic necrotic region contributes to tolerance to ROS-inducing treatments and plays an important role in therapy resistance, aggressiveness, and relapse (Vaupel and Mayer, 2007; Sattler et al., 2010). Several other mechanisms could contribute to therapy resistance, including a paradoxical increase in mitochondrial ROS production during hypoxia through a HIF-1 regulation loop (Murphy, 2009).

Brain tumors have a distorted redox homeostasis, resulting in the stimulation of survival pathways that would facilitate tumor growth and resistance. The current treatment is based on surgical excision associated with the combination of radio- and chemotherapy, but tumor recurrence remains constant with the acquisition of resistance by activation of several systems, some of which modify the redox equilibrium. Relapse occurs in part due to the redox-induced dedifferentiation of non-GSCs that would add to this therapy resistance (Bao et al., 2006; Diehn et al., 2009).

Quite a few other mechanisms could be responsible, including OS, which would modulate the efficacy of treatments and resistance in various ways, impacting on drug sensitivity, apoptosis, angiogenesis, or inflammatory pathways (Nathan and Cunningham-Bussel, 2013). GBM therapies induce the activation of redox-sensitive transcription factors, including nuclear factor-κB (NF-κB), nuclear factor erythroid 2 p45-related factor 2 (Nrf-2), or HIF-1 that would up-regulate cell survival molecules belonging to the BCL-2 family of proteins and the Akt survival pathway.

It has been shown that, during chemotherapy, tumor cells could overcome drug-induced OS by enhancing their antioxidant systems as well as an increasing P-gp pump efflux to acquire a new redox balance due the increased ROS accumulation and antioxidant systems in a process of redox rearranging (see Figure 1). This adjustment to OS would result in low levels of mitochondrial ROS, increased mitochondrial respiration, and resistance to chemotherapy (Oliva et al., 2011). Redox reactions initiated by therapy induce significant changes in cells, pathways regulating survival, and inflammation, as well as induce an up-regulation of antioxidant enzymes, thereby promoting conformational changes in the drug transporters. The redox status of the cell has a large effect on disulfide bond formation, protein folding, and dimerization of multidrug resistance (MDR) proteins such as MDR-1 (Liu et al., 2016), thereby increasing drug resistance (Tivnan et al., 2015).

IR likewise causes charged particles (electrons or ions) to traverse the cell and directly ionize DNA, causing double-strand breaks (DSB), which could be restored by homologous recombination (HR) and non-homologous end joining (NHEJ) mechanisms, or a base damage and single-strand breaks (SSB) repaired by BER and SSB repair mechanisms, respectively (Maier et al., 2016). The principal consequence of ionizing radiation is the elimination of cells predominantly by the induction of DNA damage, resulting in a diminution of cell population and a consequent functional deficiency. Radiation-induced DSB characterize the most deadly form of DNA damage, resulting in cell death if not repaired (Vlashi et al., 2011; Lan et al., 2012). Radiotherapy, associated with chemotherapy in GBM, also acts indirectly, producing free radicals that are derived from the ionization or excitation of the water constituent of the cells, resulting in the formation of aqueous free radicals and ROS, including ${\text{O}}_{2}^{\cdot -}$ and H_2_O_2_. However, these radicals are less significant than OH^·^ in producing the fatal DNA damage (Agostinelli and Seiler, 2006). Free radicals activate cell death pathways, with the production of ROS leading to massive OS, DNA damage, alterations in mitochondrial function, stem cell enrichment (Vlashi et al., 2011; Lan et al., 2012), and modifications in radio- and chemo-sensitivity (Ke et al., 2014). The adaption of tumor cells to survive could rise from cellular reprogramming (Lagadec et al., 2012) and/or the dedifferentiation of certain tumor cells to more pluripotent states, along with GSC selection among tumor cells.

During tumorigenesis, the microenvironment is in continual transition with alteration in hypoxia, nutrients, and changes in acidic stress; the result of which is that cells have to continually modify their metabolic pathways (Vander Heiden and DeBerardinis, 2017). This metabolic stress promotes the emergence of CSCs. Under normoxic conditions, glucose provides acetyl-CoA, which condenses with oxaloacetate to form citrate. Glutamine would participate in this production via α-ketogluterate. Under hypoxia or glucose deprivation, glutamine would become the major source of citrate, and tumor cells would be able to maintain cell proliferation despite a marked reduction in citrate production from glucose. Two types of GSCs are present in GBM; the first are highly proliferative cells found in the perivascular zone, and the second group is composed of quiescent cells found in the hypoxic regions, suggesting that the microenvironment could influence the metabolism of these cells (Brooks and Parrinello, 2017). GSCs depend on glycolysis to a lesser degree than more differentiated tumor cells (Vlashi et al., 2011). In addition, glycolysis was demonstrated to be greater in mesenchymal GSCs than in proneural GSCs, suggesting a correlation between the metabolism in GSCs and the tumor subtype (Mao et al., 2013). This could be due to the over-expression of the aldehyde dehydrogenase (ALDH) family of genes, especially ALDH1A3 that was markedly increased in mesenchymal GSCs (Mao et al., 2013). Furthermore, Shibao et al. have shown that the heterogeneous metabolism in GSCs could be due to environmental factors. There seems to be plasticity in the metabolism of GSCs, with a reversible switch between glycolysis and oxidative phosphorylation depending on the availability of oxygen and, as such, influenced by hypoxia. There appears to be a dichotomy suggesting that more glycolytic GSCs are found in hypoxic niches, while GSCs having an oxidative phosphorylation metabolism were found to be more in the perivascular regions (Shibao et al., 2018). Higher mitochondrial activity in GSCs would result to an increase in ROS concentrations. Furthermore, PRDX4 negatively regulates OS levels in order to protect GSCs from cell death and increase resistance to treatment (Kim et al., 2014). It has been shown that TRAP1 modified mitochondrial respiration and reprogrammed cellular metabolism (Yoshida et al., 2013). Park et al. (2019) demonstrated that an interaction in GSCs between the mitochondrial chaperone TNF receptor-associated protein 1 (TRAP1) and the major mitochondrial deacetylase sirtuin-3 (SIRT-3) improved the deacetylase activity of SIRT-3 and consequently reduced ROS production by SOD-2, the deacetylation activity of which increased. Finally, the increase in the mitochondrial respiratory capacity and the reduction in ROS production would aid GSCs to adapt to stress, thereby resisting cell death (Park et al., 2019). This distinction is reinforced by the data of Jin X. et al. (2017), which demonstrated that the vascular regions in GBM showed a proneural phenotype and would contain GSC-activated EZH2, while the hypoxic regions showed a mesenchymal profile with GSCs expressing BMI1. Both BMI1 and EZH2 promoted cell survival under stress, and EZH2 contributes to resistance to radiation and chemotherapy, suggesting that both BMI1 and EZH2 may allow plasticity of the state under environment conditions.

## The Pathways of Resistance to Radio- and Chemotherapy and Production of ROS Are Interconnected to Ensure Survival

IR initiates alterations in oxidative phosphorylation, which would augment the glycolytic rate that is linked to radio-resistance. An increase in ROS would induce HIF-1α stabilization (Dewhirst et al., 2008) and pyruvate dehydrogenase kinase 1 (PDK-1) (Rademakers et al., 2008) that would act to limit the entry of pyruvate into the Krebs cycle, thereby decreasing mitochondrial oxygen consumption (Papandreou et al., 2006).

It has also been shown that human biliverdin reductase, which converts biliverdin to bilirubin, a potent antioxidant responsible for the maintenance of intracellular redox homeostasis (Sedlak and Snyder, 2004), was induced by OS associated with hypoxia and ROS induced during TMZ treatment (Kim et al., 2013). GBM radio-resistance is linked to mitochondrial modifications and an unusually high production of ROS scavengers. An increase in mitochondrial H_2_O_2_ induces survival mechanisms with an up-regulation of catalase and mitochondrial superoxide dismutase (SOD-2) (Lee et al., 2004). An elevated activity of the latter enzymes and an increase in the level of reduced glutathione (GSH) are associated with the acquisition of resistance (Trachootham et al., 2009; Ortega et al., 2011). The interconnection of the consequences of the two types of therapeutic strategies is illustrated by the fact that GSH can form glutathione S-conjugated molecules to enable drug efflux by MRP-1 (Krause et al., 2007), and higher concentrations of GSH result in elevated chemotherapeutic resistance in numerous cancers (Traverso et al., 2013). TMZ is an alkylating agent, which functions by methylating guanine in the DNA at the O6 position, preventing a match with thymine in the replication cycle (Jiang et al., 2012). TMZ induced DNA damage and the production of ROS (Lo Dico et al., 2019), but the efficiency is very relative since nine patients out of 10 relapsed (Stupp et al., 2009). TMZ resistance is under the control of the DNA repair enzyme, O6-methylguanine-DNA methyl-transferase (MGMT). The expression of MGMT is silenced by methylation in the promoter region of the gene in half of all GBM patients (Hegi et al., 2004). Rocha et al. (2016) showed that TMZ resistance and recurrence are associated to OS. Besides its alkylating effect, TMZ has other cellular functions, including cell death, carried out by increasing the level of ROS (Zhang et al., 2010) and controlling autophagy (Yan et al., 2016), apoptosis (Roos et al., 2007), and HIF-1α activity (Lo Dico et al., 2018). There is a close relationship between autophagy and TMZ resistance since TMZ toxicity depends on both (i) the chaperone protein folding function and (ii) the augmentation of protein degradation pathways, both of which are mediated by the ER and autophagy (Lo Dico et al., 2019).

Chang et al. (2017a) showed that specificity protein-1 (Sp1), a nuclear transcription factor, protects GBM cells against stress and TMZ-induced death. Sp1 has been shown to up-regulate antioxidant genes, especially those that are beneficial against stress-induced cellular damage (Yeh et al., 2011; Chang et al., 2017a). Poschmann et al. (2015) linked the peroxiredoxin-1 (PRX-1) status in glioma to OS caused by therapy and showed that a decreased level of PRX-1 was associated with a better response to chemotherapy (Dittmann et al., 2012). It was hypothesized that PRX-1 (Svendsen et al., 2011) and thioredoxin (TRX) (Saitoh et al., 1998), when bound to apoptosis signal-regulating kinase 1 (ASK1), inhibited their phosphorylation and the subsequent activation of the cell death pathways that follow their dissociation under OS. As such, the overexpression of PRX-1 generated by therapy in GBM, serves as a survival factor and protects against therapy-induced stress (Svendsen et al., 2011). The level of expression of peroxiredoxin-2 (PRX-2) correlated with the resistance to radio- or chemotherapy in GBM (Park et al., 2000) and the overexpression of TRX, particularly in the hypoxic region of tumors, contributing to chemotherapy resistance, which was negatively regulated by TRX-interacting protein (TXNIP) (Haas et al., 2018). The TXNIP level is directly correlated with patient survival in GBM (Zhang et al., 2017), and increasing levels of TRX-1 escalate the scavenging of ROS generated by various anticancer agents (Marks, 2006). In the brain, GSH and GSH-related enzymes, glutathione peroxidase (Gpx1), are essential for the elimination of ROS and detoxification (Traverso et al., 2013). Zhu et al. (2018) demonstrated that TMZ-resistant glioma cells have higher levels of glutathione reductase (GR) and GSH than TMZ-sensitive cells. Dalavaikodihalli Nanjaiah et al. (2019) showed that GSH, GR, and catalase were all up up-regulated through the glutamate-mediated activation of N-methyl-D-aspartate receptor. The expression of the amino acid antiporter that mediates the exchange of extracellular cysteine and intracellular glutamate across the plasma membrane, the system xc^−^, was found to be elevated in GBM cell lines, increase GSH production, mitochondrial biogenesis, oxidative phosphorylation, and ATP generation (Polewski et al., 2016), and conferred resistance to OS while decreasing sensitivity to TMZ.

## ROS Production and Inflammation

Inflammation induced by GBM therapies participates in the activation of tumor cell death processes through the production of ROS, with different pathways counteracting the effects of chemo- and radiotherapy. These pathways include Nrf-2/Kelch-like ECH-associated protein 1 (Keap1), mitogen-activated protein kinases (MAPKs), nuclear factor kappa B (NF-κB), protein kinase C (PKC), signal transducers and activators of transcription-3 (stat-3), and peroxisome proliferator-activated receptor-γ (PPARγ), which regulate the antioxidant defense systems (Jaramillo and Zhang, 2013). Indeed ROS, OS pathways, and inflammation are all closely linked and participate in the resistance to therapy. ROS and RNS production is central to the progression of inflammation (Mittal et al., 2014). ROS acts as both a signaling molecule and a mediator of inflammation (Conti et al., 2010). Depending on the level of ROS induced and the initial resistance capacity of tumor cells, the associated inflammation can have an important impact on the fate of the cell. The NF-κB a key regulator of inflammatory gene expression (Conti et al., 2007) has been shown to stimulate, via transcription, genes encoding pro-inflammatory cytokines (IL-6), cell adhesion molecules, inducible nitric oxide synthase or iNOS (NOS2), and cyclooxygenase-2 (COX-2) (Grivennikov et al., 2010) and, together with NO derived from iNOS and PGE_2_, has key functions in the pathogenesis of inflammation and carcinogenesis (Nagai et al., 2002). The connection between NF-κB signaling and ROS is complex. Depending on the circumstances, ROS can either activate or inhibit NF-κB signaling and, as such, interact with NF-κB signaling pathways in many ways. NF-κB activity is regulated by the expression of ROS and NF-κB-dependent genes that affect the concentration of ROS in the cell (Morgan and Liu, 2011). NF-κB activation can also contribute to the protection against high levels of ROS produced during therapy by positively regulating manganese SOD-2 (Djavaheri-Mergny et al., 2004; Dhar and St Clair, 2012), copper–zinc superoxide dismutase (Rojo et al., 2004), ferritin heavy chain (Pham et al., 2004), TRX-1 and TRX-2 (Djavaheri-Mergny et al., 2004; Kairisalo et al., 2007), glutathione S-transferase π (GST- π), and glutathione peroxidase-1 (Gpx1) (Schreiber et al., 2006) in response to OS (and hypoxia). Heme oxygenase (HO-1) is also up-regulated by NF- κB (Lin et al., 2007).

However, enzymes that stimulate the production of ROS and their targets are all up-regulated, including NADPH oxidase (NOX-2) (Anrather et al., 2006) and xanthine oxidoreductase (XOR), which are regulated by NF-κB (Xu et al., 1996), iNOS and NOS1 (Nishiya et al., 2000), COX-2, and prostaglandin G/H synthase 2 (Annabi et al., 2009). The latter plays a key role in inflammation (Smith et al., 1996) and COX-2 by stimulating the release of pro-angiogenic prostaglandins (Tsujii et al., 1998), acting on tumorigenesis and tumor growth. Annabi et al. (2009) showed an enhanced COX-2 expression in the CD133^+^ GSC population.

With the induction of inflammation, there is an augmentation in Il-6 production, which would constitute a pro-survival signal in GBM (Van Meir et al., 1990) and particularly in GSCs, which preferentially express the IL-6 receptor alpha (IL-6Rα) and glycoprotein 130 (gp-130), generating the heterodimerization of receptors and culminating in the activation of stat-3 signaling (Wang et al., 2009). Tamari et al. (2017) explored the control of ROS by IL-6 in radio-resistance in GBM cell lines and showed that IL-6 was implicated in the inhibition of mitochondrial ROS (O2^·−^) and intracellular ROS (^·^OH, ONOO^−^). stat-3 has also been demonstrated to be a central element linking extracellular signals to transcriptional pathways involved in proliferation and cell cycle progression (Brantley and Benveniste, 2008).

During therapy in GBM, stress, inflammation, and pro-apoptotic signals could also lead to the activation of the Nrf-2 pathway. Nrf-2 maintains redox homeostasis (Ma, 2013) and is an essential antioxidant transcription factor regulator in cells against xenobiotics capable of triggering DNA damage and initiating carcinogenesis (Kensler et al., 2007). However, the role of Nrf-2 in drug resistance was suggested by its action to induce antioxidant enzymes (Wang et al., 2008). Indeed elevated levels of ROS (during radio- or chemotherapy) induced the translocation of Nrf-2 to the nucleus, where it bound to the antioxidant response element (ARE) or the electrophile-response element in the promoter region of Nrf-2, targeting antioxidant and anti-apoptotic genes, including heme-oxygenase 1 (HO-1) (Pan et al., 2013; Tebay et al., 2015), which catalyzes heme degradation and the production of carbon monoxide (CO), ferrous iron (Fe^2+^), and biliverdin (Kim et al., 2013). HO-1 plays a protective role in chemo-resistance through the induction of autophagy MAPK kinase pathways and protection against ROS damage to increase the resistance to therapy (Johnson, 2015). The shift between activation and inactivation of Nrf-2 protects GBM cells from the deleterious effects of ROS in cells produced by therapy, thereby preventing apoptosis and sustaining cell survival (Ma, 2013). It has been shown also to support tumorigenesis in primary cultures of GBM cells by promoting proliferation and resistance to cell death programs such as ferroptosis (Fan et al., 2017). Nrf-2 plays a transcriptional regulatory role on the ECM remodeling marker MMP-2, favoring chemo-resistance (Rajesh et al., 2019).

The translocation of Nrf-2 to the nucleus caused by OS has been shown to activate the glutamate cysteine ligase modifier subunit, glutathione S-transferase π, GSH availability and use, glutathione reductase, and glutathione peroxidase (Sporn and Liby, 2012; Rocha et al., 2016). Besides the Nrf-2 pathway, other highly efficient antioxidant defense systems, the PRXs, have a key function in the preservation of cellular redox homeostasis, preventing the oxidation and aggregation of proteins. PRXs are activated during therapy and, as such, play an essential role in therapy resistance (Sharapov and Novoselov, 2019).

## ROS and Autophagy

Numerous studies have shown that TMZ could induce autophagic cell death in GBM cell lines that were under the control of Nrf-2 (Kanzawa et al., 2004; Stepkowski and Kruszewski, 2011; Zhou et al., 2013). The induction of stress by therapy could affect tumor cells in different ways: including the induction of autophagy, cellular senescence, apoptosis, or necrosis, all of which are, in part, interconnected, and damages induced by therapy—either chemo- or radiotherapy could induce autophagy as a housekeeping process (Kanzawa et al., 2004; Lin et al., 2012). Autophagy could also prevent the initiation of tumorigenesis or, inversely, autophagy could support gliomagenesis by increasing cancer cell survival under unfavorable conditions. Glioma cells can escape to stress-induced effects with autophagy to survive some therapies (Kim et al., 2017). However, this process is complex because massive autophagy can induce lethal “self-eating” and apoptosis (Li et al., 2011). Autophagy, which induces the degradation and recycling of long-lived proteins or organelles, can be directed by ROS and so with being indirectly regulated by antioxidant systems. Autophagy is an essential event in GBM resistance to treatment and presents a double face.

Several pathways are associated with its activation in GBM cells; the extracellular signal-regulated kinase1/2 (ERK1/2) pathway (Scherz-Shouval et al., 2007), class I phosphatidylinositol 3-phosphate kinase (PI3K)/AKT/mammalian target of rapamycin (mTOR) pathway (Fan et al., 2010), and nuclear factor kappa-B (NF-κB) pathway. The redox imbalance has an essential role in the process, with the mitochondria as the main source of ROS in autophagy signaling. Through these pathways, dual effects of TMZ on autophagy exist, depending on the concentration. Both autophagy inducing death and protective autophagy can be achieved, and this would affect tumor cell death, GSC differentiation, or resistance to treatment (Buccarelli et al., 2018; Feng et al., 2019).

It has been suggested that, during induced stress, there is a simultaneous activation of autophagy/mitophagy and apoptosis (Kubli and Gustafsson, 2012). Mitophagy, a selective autophagic process, is normally an onco-suppressor process that prevents oncogenic transformation (Wang and Klionsky, 2011). During cancer cell survival under cytotoxic stress, these stresses could induce damage in the mitochondria, resulting in the removal of the damaged mitochondria from the cell by mitophagy and thereby reducing mitochondrial ROS. If mitochondrial damage is not repaired and mitophagy is inactivated, the apoptotic pathway would be activated, resulting in cell death (Kubli and Gustafsson, 2012). However, transfer of the mitochondria between cancer-associated fibroblasts and a GBM tumor cell subpopulation could help cells to escape to this process (Salaud et al., 2020).

## Impact of ROS and Glioblastoma Stem Cells

OS or other stresses such as hypoxia result in the enrichment of GSCs (Pistollato et al., 2010). GSCs have a radio- and chemo-resistant phenotype responsible for the constant relapse (Liu et al., 2015), in part because of the high expression of anti-apoptotic proteins and drug efflux transporters (Nakai et al., 2009) and the constitutive activation of the DNA repair element, poly-ADP-ribose polymerase 1. MacLeod et al. (2019) explored the mechanisms of TMZ sensitivity in GSCs and confirmed the implication of key members of the mismatch repair (MMR) pathway, including MutL homolog 1 (MLH1), MutS protein homolog-2 and−6 (MSH-2 and MSH-6), and the MMR endonuclease PMS2, which plays an important role in mediating oxidative DNA damage repair (Brierley and Martin, 2013). The DNA replication licensing factors MCM-8 and MCM-9, which together form a dimeric helicase complex, are likewise involved in homologous recombination with the zinc finger CCCH domain-containing protein 7A, ZC3H7A, the inactivation of which results in an increased sensitivity to oxidative DNA damage repair (MacLeod et al., 2019). This resistance can be attributed, among others, to the detoxification mechanisms of O2^·−^ and NO formation and enhanced autophagy (Lyakhovich and Lleonart, 2016). Baulch et al. (2016) demonstrated that OS induced by radiation in primary GBM cell cultures resulted in the secretion of extracellular vesicles (EVs) that induced cellular reprogramming to induce pluripotency and were amplified with repeated irradiations. Similarly, mesenchymal transdifferentiation and radio-resistance in GSCs could be triggered by the activation of NF-kB (Bhat et al., 2013). Resistance to therapy and tumor recurrence are amplified by the ROS-induced Nrf-2 activity and the consequential maintenance of GSC self-renewal and proliferation that would result in tumor relapse (Zhu et al., 2013) and inhibition of GSC differentiation (Zhu et al., 2014).

The expression of stemness markers has been shown to increase with inflammation, hypoxia, or radio- and chemotherapy (Hsieh et al., 2015). The CD133^+^ and Bmi-1 proteins associated with stem cells and drug resistance are linked to increased SOD-2 expression (Siddique and Saleem, 2012). SOD-2 overexpression in GSCs reduces the O2^·−^ reaction and caspase-dependent apoptosis, culminating in the acquisition of TMZ resistance (Chien et al., 2019). Sp1, which modulates SOD-2, contributes to the tolerance of TMZ in GBM cells (Chang et al., 2017a,b). Sp1 also promotes p53-induced glycolysis regulatory phosphatase expression in GBM by decreasing OS in cells through the pentose phosphate pathway-mediated NADPH generation, an important ROS scavenger in cells (Tang and He, 2019).

Additionally, IR augmented the transdifferentiation of GBM cells, in particular, that of GSCs into vascular endothelial cells (Soda et al., 2011; Deshors et al., 2019) that resulted in improved neovascularization in GBM tumor and thus participated indirectly to patient relapse. Furthermore, H_2_O_2_-induced OS selectively increased miR-34a (Baker et al., 2016), which also triggered the transdifferentiation of GSCs into vascular endothelial cells (Jin Z. et al., 2017). SOX-2 and stat-3 have been shown as modulators of these actions (Smith and Macleod, 2019). In this context, radio- and chemo-resistant GSCs present a metabolic adaptation presenting a reduced glucose dependence, an improved lipid catabolism, ROS, mitogen-activated protein kinases (MAPKs) activity, and NAD^+^ level, and an amplified SIRT1/PGC1 axis that promotes autophagy, resulting in an increase in the maintenance and repair machinery (Ye et al., 2013).

## ROS and Tumor Microenvironment

It has been suggested that stress stimuli from the microenvironment maintain the GSC subpopulation, which has a high level of drug resistance. Microenvironment is, in fact, essential for tumorigenesis and dysregulation of redox equilibrium.

The tumor microenvironment represents non-tumor cells within the tumor, which include the inflammatory infiltrate predominantly of microglia and macrophages, tumor-infiltrating lymphocytes, neutrophils, normal and reactive astrocytes, cancer-associated fibroblasts (CAFs), endothelial cells, and vascular pericytes (Bissell and Radisky, 2001; Tlsty and Coussens, 2006). The TME also contains proteins and non-protein biomolecules (polysaccharides, hormones, NO, etc.) that make up the extracellular matrix (ECM). The TME is principally perceived and confirmed in niches, however, it plays an important role in regulating everything in the tumor and in the surrounding tissue (Schiffer et al., 2018). Indeed, interactions between the tumor cells, ECM, soluble factors and blood vessels generate an intricate diverse environment that is in continual transformation supporting and maintaining tumorigenesis (Greaves and Maley, 2012). The metabolism of the different cells present in the TME, cell-cell interactions, the remodeling of ECM proteins to form the structure of the TME and the blood supply, give raise to several structural environmental factors such as hypoxia (O_2_ tension varying from 0.1 to 3%), acidity and alterations in the composition of the ECM and the accretion of soluble factors including O_2_, nutrients, ROS, RNS, ATP, Ca^2+^, H^+^, growth factors, chemokines and cytokines (Frisch et al., 2019). All these factors have an effect on the metabolism of cells and therefore, on the function of the cells, thus the TME is constantly fluctuating and plays a critical role in drug resistance, angiogenesis, cell death, DNA repair, OS, immune escape, the level and activity of multidrug resistance (MDR)-related genes, tumor progression, and epithelial-to-mesenchymal transition (Zhang et al., 2020). As such, the TME represents a key factor in defining and regulating the preservation of tumor heterogeneity, tumor progression, and drug resistance (Da Ros et al., 2018). The principal function of the relationship between tumor cells and TME in tumorigenesis has been said to be the dynamic collaboration between the two to stimulate the proliferation and protection of the tumor cells from immune surveillance and radio- and chemotherapy. Indeed it has been shown that most of the non-tumor cells present in the TME assume a tumor-promoting phenotype subsequent to alterations by the local environment on their cell functions, which include changes in gene expression.

ROS also affect cells that constitute the TME. OS in the TME is one of the main factors that mediate the conversion of cell types, such as normal fibroblasts and mesenchymal stem cells to CAFs, which play a key role in tumor cell proliferation, survival, angiogenesis, invasion, inflammation, and ECM remodeling via cross-talk with cancer cells through paracrine signals (Costa et al., 2014; Salaud et al., 2020). In addition to regulating the conversion of fibroblasts to CAFs, the oxidative TME can also increase the production of paracrine signals and matrix remodeling enzymes that would promote the invasion and metastasis of tumor cells. The regulation of protein tyrosine phosphatases (PTPases) by ROS appears as a key mechanism capable of regulating signaling by cell surface receptors, including tyrosine kinase receptors and integrins. For instance, an increase in ROS by CAFs causes the secretion of pro-invasive signals, including HGF, IL-6, VEGF, CXCL-12, and CXCL-14. ROS activation of the CXCL-12/CXCR-4 signaling pathway contributes to a cross-talk between tumor cells and CAFs (Orimo et al., 2005). For example, the cytokine CXCL-12 is secreted by CAFs, while its receptor CXCR-4 is found mainly on tumor cells. Thus, CXCL-12 signaling by receptor CXCR-4 requires an intimate interaction between CAFs and tumor cells, which would result in the proliferation of tumor cells as well as acceleration of neo-angiogenesis due to the recruitment of endothelial progenitor cells (Costa et al., 2014). Thus, chemokines released by non-tumor cells can act as paracrine factors, creating a communication with tumor cells to promote tumorigenesis (Coppé et al., 2010; Fiaschi and Chiarugi, 2012). CAFs and/or cancer-associated macrophages (CAMs) collaborate together to engender a pro-oxidant environment. Due to the activation of NOS2, CAMs can actively fabricate ROS, which will instigate the recruitment of CAFs and the activation of MMPs (Giannoni et al., 2012). The chemo-attractant, stromal-derived factor-1α (SDF-1α)/ hemokine (C-X-C motif) ligand 12 (CXCL-12), the C-X-C receptor type 4 (CXCR-4), and the cysteine protease cathepsin K (cat K) are localized to GSC niches in GBM (Hira et al., 2015) and SDF-1α acting through its interactions with CXCR-4 and/or its second receptor CXCR-7 on GSCs facilitates the homing of GSCs to niches, while cat K, which is up-regulated by ROS (H_2_O_2_) (Tsai et al., 2014), can cleave and thereby inactivate SDF-1α and, in doing so, facilitate the migration of GSCs out of the niches.

MDSC, Treg, and CAM provide an immunosuppressive environment that would contribute to tumor cell proliferation, invasion, and resistance to chemotherapy (Badie and Schartner, 2000; Beier et al., 2012; Hira et al., 2017). CD8^+^ T cells are crucial for the anticancer immune response in tumors; however, the immunosuppressive environment formed in the TME would ultimately result in the suppression of the cytotoxic T lymphocyte response, culminating in cancer progression. Inflammatory cells support tumor growth, invasion, and therapy resistance instigated by the secretion of specific molecules and factors that favor the anti-inflammatory activity (TGF-ß, ARG1, and IL-10), tissue remodeling, and angiogenesis (VEGF, MMP2, MMP9) (Grivennikov et al., 2010). Furthermore, the effect of inflammatory cells on the ECM to release MMP would directly affect GSCs. High levels of ROS are major factors in immune-suppression and inhibition for T cell activation and proliferation, while low levels of ROS generate T cell activation in the TME, whereas CAFs have been implicated in immune-suppression of CD8^+^ T cells in GBM. Ford et al. (2020) have shown that the pharmacologic inhibition of NADPH oxidase 4 in the proneural subtype of GBM “normalized” CAFs to a quiescent phenotype, resulting in intratumoral CD8^+^ T cell infiltration and thereby overcoming the CAF-mediated CD8^+^ T-cell exclusion effect. CAFs or CAMs would together synergize to generate a pro-oxidant environment.

The redox landscape extends beyond the single cell to the TME. Moreover, the TME is associated with a reduced oxygen concentration, initiating a hypoxic environment that is linked to an amplified tumoral aggressiveness (Narayanan et al., 2020). Hypoxia would also influence intercellular communication by varying the release and the uptake of EVs by the cells. Studies have shown that hypoxia-derived tumor EVs play an important role in gliomagenesis (Kore et al., 2018). Exosomes are small EVs that transport cytosolic biomolecules, such as miRNAs and proteins, from virtually all cells in the body to neighboring and distal cells via the endocytic pathway. Recently, EVs have received a significant interest as transporters of biological mediators and have shown to be an important messenger in the intercellular communication between the tumor and the TME (Sullivan et al., 2017). Tumor cells exclude EV to engage non-tumor cells present in the TME and reprogram these cells from their normal activity to be more pro-tumorigenic (Whiteside, 2016). The transfer of molecules via EVs has emerged as a key messenger in intercellular communication in the TME. These EVs transport a diverse selection of molecules, including proteins, lipids, or nucleic acid cargoes. Tumor cells exclude EVs to the non-tumor cells, resulting in molecular, transcriptional, and translational modifications that cause these cells to fabricate factors required for tumor growth and at the same time alter the function of these cells (Santos and Almeida, 2020). TME stromal cells would, in turn, generate their own EVs containing and transferring molecules not only to the tumor but also to other cells in the TME, enhancing their pro-tumorigenic activity (Kalluri, 2016). Thus, EVs are able to propagate and maintain the TME as well as regulate the redox environment. An imbalance in the redox status would also alter the quantity of exosomal cargo proteins and consequently influences the redox levels in the EV-receiving cells. For example, the redox-sensitive signaling pathway PI3K/Akt/endothelial NOS controls the exosomal release of angpoietin-2 that has a key function in the remodeling of tumor vascularity.

Besides facilitating the transformation of stromal cells, the oxidative TME plays a key role in the output of paracrine signals and matrix remodeling enzymes that directly affect tumor cells, resulting in proliferation and invasion. In this context, the role of redox regulation by the PTPases has emerged as a key signal-regulating mechanism (Frijhoff et al., 2014). Acidosis, usually associated with high concentrations of lactate, presents a crucial stress factor in TME and would affect the comportment of the tumor. Tumorigenesis leads to alterations in metabolism, resulting in an amplification in glycolysis, caused by aerobic glycolysis or the “Warburg effect,” which triggers a prompt supply of energy that is correlated to an increased conversion of glucose to lactate (Gatenby and Gillies, 2004; Vander Heiden et al., 2009; Vaupel and Multhoff, 2017). Lactate has been shown to be associated with tumor progression, therapy resistance, and immune escape (Brizel et al., 2001; Sattler et al., 2010; Kahlon et al., 2016). Radiotherapy resistance could also be intensified by the antioxidant properties of lactate, which would neutralize the ROS produced by IR to cells. Moreover, studies have shown that acidosis supports cell motility and migration as well as the degradation and remodeling of the ECM (Goetze et al., 2011). Lactate has been implicated in tumor angiogenesis and the expression of GSC markers (Hjelmeland et al., 2011). Exposure to acidic conditions could result in autophagy, which, as stated earlier, is connected with the preservation of the GSC phenotype and resistance to therapy (Lomonaco et al., 2009; Peppicelli et al., 2017). Acidosis could also neutralize ROS associated with radiotherapy, inhibit radiation-induced apoptosis, enhance the activity of P-glycoprotein (P-gp), and/or reduce the rate of proliferation of tumor cells (Peppicelli et al., 2017). In addition, acidosis would reduce the immune response by affecting the infiltration of tumor cells and the cytokine release by T cells, impeding monocytes, blocking the cytotoxic activity of natural killer (NK) and CD8^+^ T cells, and boosting the activity of MDSC (Vaupel and Multhoff, 2017).

Thus, high levels of ROS present in tumor cells could result from either an amplified metabolic activity, mitochondrial dysfunction, peroxisome activity, deregulated cell receptor signaling, oncogene activity, enhanced activity of cyclooxygenase, lysyl oxidase, and thymidine phosphorylase, or communication with the immune infiltrate (Babior, 1999; Storz, 2005), suggesting that ROS and RNS can support numerous facets of tumor development and progression.

## Pivotal Function of Antioxidant in Glioblastoma: Effect of Nutrition

It is now accepted that the synergistic effects of active compounds present in fruit and vegetables are responsible for their anticancer actions, and studies have shown a reverse effect of a diet rich in antioxidants in GBM (Chen et al., 2002; Tedeschi-Blok et al., 2006). Numerous studies have suggested that modulation of metabolism and ROS production by specific natural dietary constituents, such as phytoestrogens, flavonoids, polyunsaturated fatty acids, and vitamins, may have a protective action against cancers.

We used an ethylnitrosourea (ENU)-induced malignant GBM pregnant rat model developed from Koestner et al. (1971) to explore the influence of nutrition on gliomagenesis using an experimental diet (PtcD) composed of different constituents suggested to interfere in carcinogenesis, mainly phytochemicals, and its effects were compared to a diet without the phytochemicals (StD). In male rats fed the PtcD, the frequency of GBM was clearly diminished compared to that of rats fed the StD; however, in females, the outcome was negligible. An evaluation of the gene expression of proteins implicated in proliferation, apoptosis, and response to OS in male brain tumors depicted that the inhibition of the systemic effects (loss of body weight and liver mass, plus reduced liver mitochondria mass) was linked to an increase in Bcl-2 and catalase and a decrease in Ki-67, SOD-1, and SOD-2 (Pouliquen et al., 2008). These data suggested that the degree of aggressiveness of GBM could be controlled by dietary interventions and recommended that some phytochemicals with antioxidant properties could participate to the mechanism. We explored this hypothesis in a study using SUVIMAX-like diet (“Supplementation en Vitamines et Minéraux Antioxydants”), where rats were fed with a diet enriched with alpha-tocopherol, beta-carotene, vitamin C, zinc, and sodium selenite. We observed that this diet was associated with a considerable lag in the clinical signs of the disease but not a statistically significant difference in the incidence of glioma in an ENU model. The SUVIMAX-like diet decreased the candidate markers of tumor aggressiveness and gliomagenesis progression. The expression of the mRNA of two common markers in human glioma, SOD-2 and IGFBP5 (insulin growth factor binding protein), was reduced in the tumors of rats fed the antioxidant diet. In addition, the transcripts of two markers linked to brain tumor proliferation, PDGFR-β (platelet-derived growth factor receptor beta), and Ki-67 were also significantly decreased. Overall, our results suggested a protective role for antioxidants in restraining the aggressiveness and, to some extent, evolution of GBM in a rat model (Hervouet et al., 2013).

Studies have highlighted the protective roles of hormones (Kabat et al., 2010; Zhou et al., 2019), and vitamins (Pouliquen et al., 2008; Kyritsis et al., 2011) in GBM as well as in other cancers (Han et al., 2015). However, the implication of ROS in the observed protection remains to be investigated.

## Conclusion and Future Directions

The oncogenic activity of oxidants hinges on four principal functions in tumorigenesis. Firstly, the mutagenic potential of oxidants could be implicated in the initiation of tumorigenesis. A considerable amount of mutagenic DNA damage in cells can be attributed to endogenous ROS and RNS; two of the principal candidates being hydroxyl radical (OH^−^) and peroxinitrite (ONOO^−^), both of which have 8-oxo-guanine and single- and/or double-strand DNA breaks (Marnett, 2000). Secondly, the influence of oxidants on intracellular signaling pathways regulating cell proliferation and survival would promote tumorigenesis (Cerutti, 1985). Indeed low doses of hydrogen peroxide (H_2_O_2_) and superoxide (${\text{O}}_{2}^{-}$) have been shown to stimulate cell proliferation in several tumors (Storz, 2005). ROS may diminish the requirement of cells for growth factors by decreasing the activation level of cognate receptor tyrosine kinase (RTK) or by trans-activating receptors in a ligand-independent manner (Rhee et al., 2000). Since RTK is linked to many downstream signaling cascades, numerous growth-related signaling actions triggered by oxidants could partially mimic the upstream activation of RTK-dependent signaling (Pani et al., 2000). Thirdly, the oxidants have impact on cell motility and invasiveness. Data suggest that tumor evolution is closely linked to the hypoxic TME in tumor lesions (Allen and Jones, 2011). The production of ROS in mild hypoxic environments could be the result of deregulation of mitochondrial respiration (Klimova and Chandel, 2008). Finally, the role of oxidants in stromal reactions is necessary for tumor progression and dissemination, such as inflammation/repair and angiogenesis (Comito et al., 2011). Indeed hypoxia that arises at the start of tumor growth and produces cell necrosis results in the activation of hypoxia-responsive genes in tumor and non-tumor cells. It also supports the deployment and persistence of immune cells that are mainly glycolytic, including macrophages that generate large amounts of ROS. This has huge concerns for the cells present in this niche, as they must adjust to survive in this very oxidative environment. The increased levels of ROS also stimulate pathways in leukocytes to secrete more cytokines that support tumor growth, resulting in new cellular mutations, and could transform other cells and induce apoptosis.

In the future, it would be interesting to study the impact of oxidative stress in new therapies such as immune or oncolytic virus-based therapies. Similarly, ROS production effects on radiation therapies have been described, but their radio-sensitizing modulation properties at the chemical and biological levels need to be completed. Finally, since ROS production can be partially controlled positively or negatively by nutrition and/or by pollutants, it remains to be established how environmental factors could be incorporated into treatment strategies.

## Author Contributions

CO, LO, LL, and FV wrote the first draft and the following versions. All authors contributed to the article and approved the submitted version.

## Conflict of Interest

The authors declare that the research was conducted in the absence of any commercial or financial relationships that could be construed as a potential conflict of interest.
